# Vitamin D Levels of Out-Patients in Lithuania: Deficiency and Hypervitaminosis

**DOI:** 10.3390/medicina54020025

**Published:** 2018-04-26

**Authors:** Andrius Bleizgys, Jevgenij Kurovskij

**Affiliations:** Clinic of Internal Diseases, Family Medicine and Oncology, Faculty of Medicine, Vilnius University, LT-10223 Vilnius, Lithuania; j.kurovskij@gmail.com

**Keywords:** vitamin D, seasons, sex factors, age factors

## Abstract

*Aim*: Data on the prevalence of vitamin D deficiency in Lithuania are scarce. The aim was to assess the reserves of vitamin D in different age groups of out-patients, regarding the season of the year. *Methods*: Data on serum 25-hydroxyvitamin D (25(OH)D) levels from blood tests made in 2012–2014 were obtained from one laboratory, and a retrospective cross-sectional analysis was performed. *Results*: A total of 9581 subjects were included. The mean age of the participants was 33 ± 23 years. The mean levels of vitamin D were higher in males than in females (*p* < 0.001). The highest mean 25(OH)D levels were in 0–9-year-old group, the lowest were in the 10–19-year-old group and in the group of participants that were 70 years and older (*p* < 0.001). The lowest vitamin D status was found in January, February, March, and April. The highest status was found in August and September. Overall, vitamin D deficiency, sufficiency, and hypervitaminosis were detected in 67%, 21%, and 12% of cases, respectively. Most cases with hypervitaminosis were in the group of children up to 2 years of age. *Conclusion*: Vitamin D status demonstrated clear seasonality. Significant sex-related differences of vitamin D statuses were also determined. Vitamin D deficiency was very prevalent in almost all age groups. Young children (aged up to 2 years) are of special interest for further research involving other types of 25(OH)D assays, such as those based on high-performance liquid chromatography (HPLC), since the real prevalence of “true” vitamin D hypervitaminosis in Lithuania’s children is still to be determined.

## 1. Introduction

Interest in the role of vitamin D (VitD) for human health is constantly growing. VitD appears to be essential not only for “healthy bones”, but also for many other organs and tissues [1]. For humans, the main source of VitD is the synthesis of vitamin D3 in the skin, under the influence of solar ultraviolet radiation. After two hydroxylation steps, VitD is transformed into an active form, 1,25-dihydroxyvitamin D, or calcitriol. The first step takes place in the liver and produces 25-hydroxyvitamin D (25(OH)D). The second hydroxylation is performed by renal proximal tubular epithelial cells, as well as by some extra-renal tissues and cells, such as endothelium [1]. Serum 25(OH)D levels are widely accepted as the best marker of VitD status.

Low VitD status seem to be a global problem: It was estimated that about one billion humans could have VitD deficiency or insufficiency [2]. A high prevalence of vitamin D deficiency was found in many European countries, particularly in Eastern and Central Europe [3,4,5], while much better VitD statuses were found in Norway and Sweden [6].

In Lithuania, data on the prevalence of low VitD are relatively scarce. Most studies were, however, based on small samples, but have shown a rather poor situation regarding VitD status. Gailyte et al. examined 262 men aged 19–25 years, members of the Lithuanian Armed Forces, and found that almost all of them were VitD deficient [7]. Strazdiene demonstrated that in a sample of elderly adults, only 7.9% of men and 4.9% of women had normal VitD levels (≥75 nmol/L) [8]. In the study of Zabuliene et al., 73.8% of young women (aged 16–20 years) were VitD deficient, while 22.8% had VitD insufficiency [9]. Bleizgys and Sapoka have examined young men (aged 20–39 years) twice and found that, in February or March, 96.9% of the participants had low VitD levels; however, 36.8% of them retained similar poor VitD status even in August or September [10]. Lithuania is a country located at the middle latitudes. In such areas, the intensity of solar radiation decreases significantly during the cold season, and the synthesis of vitamin D3 in the skin is reduced or almost absent during the period from October to March, and this seems to be the main natural risk factor for VitD deficiency, at least during the cold season [11,12].

In fact, larger-scale studies of Lithuanian citizens are still warranted. In this paper, we present the findings of a recent retrospective study on the prevalence of low VitD levels in various age groups and seasons, showing some quite unexpected study results.

## 2. Material and Methods

This cross-sectional retrospective study was based on the results of serum 25(OH)D levels from one private laboratory located in Vilnius (54°54′ N, 25°19′ S). The blood sera analysed in this laboratory were obtained mainly from the private primary care centres and public institutions (polyclinics), located in Vilnius city and its suburbs, as well as from some personal laboratory clients. Serum 25(OH)D levels in the laboratory are being determined using a Cobas e411 analyser (Roche; Basel, Switzerland). This analyser uses an automated immunoassay for measuring total serum 25(OH)D. In the present study, serum 25(OH)D levels below 75 nmol/L were treated as VitD deficiency, 75–118 nmol/L as sufficiency (i.e., normal VitD status), and above 118 nmol/L as VitD excess, or hypervitaminosis, according to the reference values of that laboratory. Coefficient of variance (CV) reported for repeatability of the assay was 1.7%–7.8%, and the CV of intermediate precision was 2.2%–10.7%.

For the current analysis, we used the data of the period from 2012 to 2014. If a patient had more than one analysis during the time period, then only the first sample was included for analysis. 25(OH)D levels were evaluated after having divided all participants into age groups by 10 years (0–9 years, 10–19 years, etc.), and various comparisons of 25(OH)D levels were also performed by gender and the month when the blood sample was obtained. Patient’s age was calculated at the date the blood sample was taken. Incomplete data (e.g., missing full birth-date or gender) were excluded from the final analysis.

Statistical analyses were performed using the SPSS software package (version 21.0 for Windows, SPSS Inc., Chicago, IL, USA). Nominal data are presented as frequency (percentage), and continuous data as mean ± standard deviation (SD). Continuous variables were tested for normality using the Shapiro-Wilk test. Differences of the variables between groups were analysed using Student’s *t*-test or ANOVA for variables that were normally distributed, and Mann-Whitney U test or Kruskal-Wallis test for variables with non-normal distributions, as appropriate. A *p*-value < 0.05 was considered significant.

Ethical approval: The research was carried out under permit No. 158200-14-760-274, obtained from Vilnius Regional Biomedical Research Ethics Committee. All procedures performed in the study were in accordance with the ethical standards of the institutional and/or national research committee, and with the 1964 Helsinki declaration and its later amendments or comparable ethical standards. For this type of study formal consent is not required.

## 3. Results

A total of 1196 cases were excluded due to missing data. Of the 9581 cases included in the final analysis, 6938 (72%) were women. The mean age of the participants on the date the blood sample was taken was 33 ± 23 years (range 1 month–93 years). The age group of 0–9 years and the group of 30–39 years were the largest groups, accounting for 21.7% (*n* = 2076) and 16.8% (*n* = 1605) of the cases, respectively. Unsurprisingly, the smallest number of participants composed the age groups of 70–79, 80–89 and ≥90 years (520, or 5.4%; 136, or 1.4%; and 14, or 0.2%, respectively). For that reason, all participants aged 70 years and older were further treated as one age group (70+ years of age).

Noteworthy was the fact that the popularity of VitD testing was quite unsteady throughout the year. Less frequently, 25(OH)D levels were tested in June (6.2% of all cases) and August (5.7%), while 13.9% of tests were those performed in March.

**Figure 1:**
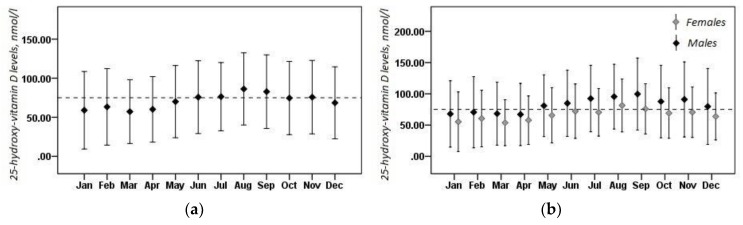
Mean serum 25-hydroxy-vitamin D levels by month: (**a**), in the overall sample; and (**b**), in females and males. Values are mean and standard deviation. The dashed line indicates the lower bound of the sufficient 25-hydroxy-vitamin D level range.

**Figure 2:**
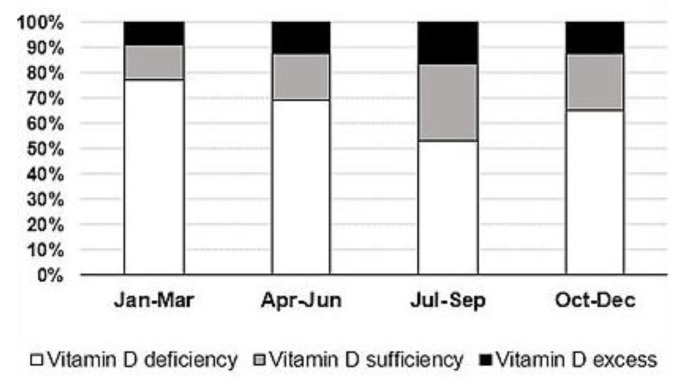
Prevalence of vitamin D deficiency, sufficiency, and excess in different seasons in the whole sample.

Mean 25(OH)D levels were 70.8 ± 9.4 nmol/L, and men had significantly higher mean 25(OH)D levels than women (81.0 ± 55.7 nmol/L and 64.8 ± 41.6 nmol/L, respectively, *p* < 0.001). A total of 6445 (67.3%) cases had low VitD levels, while only 1967 (20.5%) had normal VitD status. VitD hypervitaminosis was documented in 1169 (12.2%) cases. Low VitD levels were more prevalent in women than in men (70.6% vs. 58.6%, respectively), as well as VitD sufficiency (21.6% in women; 17.7% in men). A total of 72 cases had 25(OH)D levels greater than 250 nmol/L. Among those 72 cases, 71 were infants (32 boys) and one was a 1-year-old girl. Figure 1a presents the mean 25(OH)D levels by month. The lowest VitD status was found to be in January (59.0 ± 49.6 nmol/L), February (63.3 ± 49.0 nmol/L), March (57.1 ± 40.7 nmol/L), and April (60.1 ± 42.0 nmol/L). The highest VitD reserves have been found to be in August (86.2 ± 46.2 nmol/L) and September (82.8 ± 47.1 nmol/L). It is clear that only during the “warm” season (from June to October), mean 25(OH)D levels tend to improve until sufficiency. However, more than half of those cases tested in July–September had poor VitD status (Figure 2).

Clear seasonal changes of mean 25(OH)D levels were also detected for both males and females (Figure 1b). For each month, sex-based differences in mean 25(OH)D levels were statistically significant, except for February and April.

Table 1 presents mean serum 25(OH)D levels for each age group. By this pattern, almost all the groups were rather similar, except for group of children aged nine years and younger, which had the highest mean 25(OH)D levels. In addition, the group aged 10–19 years and the group of the oldest participants had significantly lower mean 25(OH)D levels than other groups (*p* < 0.05). All of the groups, except the group of young children (i.e., aged nine years and younger), had similar VitD deficiency prevalence-more than 70% (data not shown).

Comparisons in each age group regarding gender showed that mean 25(OH)D levels were similar between males and females, except for the group aged nine years and younger, where boys had slightly lower mean 25-OH levels than girls, and for the group aged 20–29 y, where men had lower mean 25-OH levels than women (Table 2). In contrast, in the oldest group, men had significantly higher mean 25-OH levels than women.

**Figure 3:**
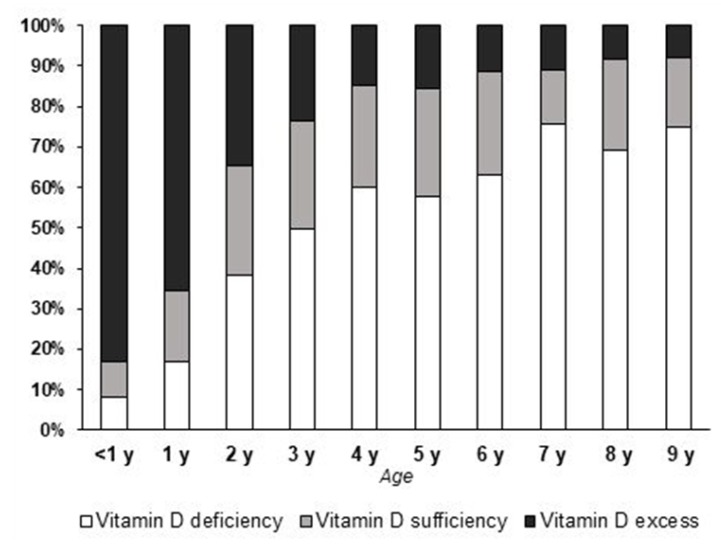
Prevalence of vitamin D deficiency, sufficiency, and excess in different age groups of children up to nine years of age.

Since the group of young children had the highest mean serum 25(OH)D levels, it was analysed more thoroughly, via sub-grouping by each year of age (Table 3). The infants and the group of 1-year-old children had significantly higher mean 25(OH)D levels than the other groups (*p* < 0.01). In addition, the majority of cases in the former groups had VitD excess, while the other groups were less different regarding the prevalence of different VitD statuses (Figure 3). Note that the group of infants, however, was the largest group in comparison to others (Table 3).

## 4. Discussion

To our knowledge, this is the first large study of VitD status in Lithuania that includes participants of a wide range of ages, and showing a high prevalence of VitD deficiency. Reduced vitamin D3 production in the skin could be the main explanation for this, since Lithuania is a country located far away from the equator, and the local climate, as well as the intensity of solar radiation, exhibits seasonal variation. At higher latitudes, vitamin D3 synthesis in the skin is almost absent in winter time, due to reduced amounts of solar UV radiation reaching the Earth and inhabitants [11,12]. As a consequence, in such latitudes, VitD status tends to exhibit a clear seasonal variation in humans [13,14]: 25(OH)D level reaches its peak from August to September, and the nadir is observed from February to March. Similar findings of seasonality were also found in our study. Low rates of vitamin D supplementation, namely, in middle-aged and older adults, may also contribute to a widespread VitD deficiency, particularly in the “cold” season. Living in a city, use of sunscreens, sun avoidance, as well as less and less time spent outdoors, may also be risk factors of a poor VitD status in the “warm” season. Lower 25(OH)D levels in the elderly might also be partly explained by the skin’s reducing capability to synthesize vitamin D3 with aging [15,16]. However, keeping in mind the retrospective nature of the present study and lack of important data, e.g., participant’s body mass index (BMI) or lifestyle habits, speculations on possible VitD status determinants should be made with caution, since, for instance, obesity is a widely-accepted risk factor for VitD deficiency. Of note, there is much controversy regarding “normal” or “optimal” serum 25(OH)D levels [17,18]. Therefore, the prevalence of VitD deficiency might have been different if another cut-off for the definition of normal 25(OH)D levels had been used for the analysis. Noteworthy, we did not use a separate category “VitD insufficiency” in our study (e.g., when the 25(OH)D level is 50 nmol/L or higher, but lower than 75 nmol/L [18]), therefore, the prevalence of “true VitD deficiency” is not clear. However, some authors argue that all levels below a certain lower limit should be referred to simply as “deficiency”, since this low nutrient status might result in many undesirable consequences, taking into account more than just “skeletal health” [19].

Men had higher mean 25(OH)D levels than women in the whole sample. Similar results were found in the Netherlands and Romania [20,21]. Although the reasons for the sex-specific differences are still unclear, possible explanations might be that women have a higher percentage of body fat, a greater avoidance of sunlight, including increased sunscreen use, and wear more covering clothing when going outside [22]. In contrast, studies in Poland and Sweden showed that women had higher mean VitD levels than men [23,24], and studies in Denmark and Germany showed no significant differences between the sexes [22,25]. It could be speculated that other factors, rather than gender itself, have greater influence on VitD status. In the present study, however, men comprised a much smaller part of sample, therefore, our results may not represent the actual differences (if any) in VitD status between males and females in Lithuania. The same could be said regarding the differences found (or not found) in each age group, since only in the youngest group (up to nine years of age) was the number of males nearly the same as the number of females. It seems that women are more likely to seek their VitD levels, and this could be explained by greater awareness of health issues and/or more health provider visits by women [26].

Unexpectedly, a high prevalence of VitD hypervitaminosis was found in the group of young children. Due to the lack of data on their nutrition, supplementation, and sun exposure, it is very difficult to find a suitable explanation for this finding. Most likely, kids having very high 25(OH)D levels might have been over-supplemented with pure VitD medication, or get high amounts of VitD from other sources, e.g., multivitamins and/or cod liver oil [27]. Genetic disturbances, e.g., when catabolism of VitD metabolites is reduced [28], are rare and do not seem to be the main explanation of the aforementioned findings. Interestingly, in one retrospective study in Romania, a small proportion of participants had 25(OH)D levels higher than 100 nmol/L, or even higher than 150 nmol/L, mainly in the group of children younger than 10 years of age [21]. It must be kept in mind that young kids have relatively low body masses and relatively high fat masses. Therefore, a large dose of pure VitD might not be unsafe for an adult, but might be harmful for a small child, due to a high concentration of VitD in blood and other tissues. In general, physicians prescribe VitD supplementation for young children more willingly, e.g., for rickets prevention and treatment, than for older children or adults (with the exception of older women, who have or are at high risk of osteopenia/osteoporosis), and sometimes very large doses are prescribed if low VitD levels are detected [29,30], or even without having first examined baseline VitD levels [31]. Over-the-counter VitD-containing supplements are also available in Lithuania, therefore, taking such supplements might also contribute to the development of VitD excess, particularly when they are being taken together with even small prescribed VitD doses [18]. However, only a small number of cases had high 25(OH)D levels that might be referred to as “undesirably high”, according to the recent recommendations [18], and, in clinical terms, this should be treated as a very positive finding.

An alternative explanation of the higher prevalence of “VitD excess” should be mentioned. C3-epimers of VitD have been found to be cross-reactive with some assays, and this could be an important confounder of VitD measurement [32]. In children, and particularly in infants, epi-25-hydroxy-D3 might compose a meaningful part of total circulating 25(OH)D (see a nice review in [33]). It was calculated that 9% of infants and 3% of adults would be misclassified as sufficient if the epimer was included in the quantification [33,34]. Based on this, we could speculate that some part of the cases in our study, namely, children up to two years of age, were overestimated as having “VitD excess”. In fact, some of the latter could be considered as having “normal” VitD reserves rather than “hypervitaminosis”, if another type of the assay, e.g., high performance liquid chromatography (HPLC), was used. Unfortunately, to our knowledge, at present, no laboratory in Lithuania is ready to offer 25(OH)D measurements using such methods as HPLC for clinical purposes.

Of note, the term VitD excess used in this study presents cases that do not necessary have excessive VitD reserves from the clinical point of view. VitD toxicity is characterized by various clinical and biochemical markers, such as elevated serum calcium levels. The retrospective nature of the study design does not allow us to retrieve such important data as serum calcium, phosphates, or parathyroid hormone (PTH) levels of the participants; we could speculate that most cases were not tested for the aforementioned parameters. Moreover, the study was not designed for such purposes, since, to the best of our knowledge, prior to the study, there were no published data on the prevalence of VitD hypervitaminosis in Lithuania’s population, on the contrary to problems such as VitD deficiency.

Undoubtedly, further large-scale studies, as well as in several regions or towns, are needed to investigate the real situation of VitD status in Lithuania, in particular, deficiency and risk for hypervitaminosis in children, and the determinants for this. Future studies should take into account the patient’s BMI, supplementation status, and other factors known to have the greatest influence on VitD status. A high prevalence of poor VitD status, however, should encourage physicians to suggest 25(OH)D level testing more frequently, and achieve a better compliance with their patients regarding proper VitD dosing according to recent guidelines [18,35].

The retrospective nature of the study, lack of data such as PTH levels, anthropometric parameters, supplementation, or lifestyle habits of the participants limit the interpretation of our findings regarding possible determinants of VitD deficiency or VitD excess. On the other hand, the large sample size and the analysis (regarding seasonality, age groups, and gender) that was performed, to our knowledge, for the first time in Lithuania on a large scale, might be named as the main strength of the present study. In addition, a high prevalence of VitD hypervitaminosis found in young children brings a novel finding to the existing data regarding VitD status in Lithuania, and should inspire further research regarding this phenomenon.

## 5. Conclusions

A high prevalence of VitD deficiency was detected in different age groups, mainly, older children and adults of various ages. In contrast, a high prevalence of VitD hypervitaminosis was found to be in young children. Large-scale studies on VitD status, also examining its potential determinants, are highly warranted for Lithuania.

## Figures and Tables

**Table 1:** Mean serum 25-hydroxy-vitamin D levels in different age groups.

Age Group, years	*n*	Median	IQR
≤9	2076	113.3 *	83.1
10–19	843	47.1 *	31.5
20–29	1172	54.7	35.9
30–39	1605	53.5	37.8
40–49	1232	51.3	36.0
50–59	1143	54.4	35.4
60–69	840	52.9	38.8
≥70	670	46.7 *	35.8

* Indicates statistically significant difference from other age groups (not marked with asterisk); Kruskal-Wallis and Mann-Whitney U tests were used; 25(OH)D, 25-hydroxy-vitamin D levels, in nmol/L; IQR, interquartile range; *n*, participants.

**Table 2:** Mean serum 25-hydroxy-vitamin D levels in age groups by gender.

Age group, years	Males			Females			*p* Value
	*n*	Median	IQR	*n*	Median	IQR	
≤9	1098	110.3	84.7	978	116.1	81.1	0.035 *
10–19	304	47.6	36.6	539	46.4	28.8	0.208
20–29	274	47.7	33.6	898	56.9	36.8	<0.001 *
30–39	315	52.1	32.4	1290	53.7	39.0	0.621
40–49	224	53.7	34.8	1008	50.7	35.5	0.157
50–59	175	54.4	35.2	968	54.5	35.4	0.456
60–69	139	54.2	39.9	701	52.6	38.4	0.654
≥70	114	52.7	31.6	556	45.6	36.1	0.002 *

* Indicates statistically significant difference between genders within each age group; Mann-Whitney U test was used; 25(OH)D, 25-hydroxy-vitamin D levels in nmol/L; IQR, interquartile range; *n*, participants.

**Table 3:** Mean serum 25-hydroxy-vitamin D levels in children aged 0–9 years, sub-grouped by age.

Age, years	*n*	Median	IQR
<1	1065	148.6 *	54.9
1	266	120.5 *	60.5
2	128	88.5	43.2
3	115	76.2	42.2
4	109	68.2	39.3
5	89	66.8	34.4
6	82	63.2	30.8
7	85	57.5	33.4
8	67	62.4	35.1
9	70	58.7	35.1

* Indicates statistically significant difference from other age groups (not marked with asterisk); Kruskal-Wallis and Mann-Whitney U tests were used; 25(OH)D, 25-hydroxy-vitamin D levels, in nmol/L; IQR, interquartile range; *n*, participants.
